# Long-Term Changes in Species Composition and Relative Abundances of Sharks at a Provisioning Site

**DOI:** 10.1371/journal.pone.0086682

**Published:** 2014-01-23

**Authors:** Juerg M. Brunnschweiler, Kátya G. Abrantes, Adam Barnett

**Affiliations:** 1 Independent Researcher, Zurich, Switzerland; 2 School of Marine and Tropical Biology, James Cook University, Townsville, Australia; 3 School of Life and Environmental Sciences, Deakin University, Burwood, Australia; 4 Oceans IQ, Cairns, Australia; Institute of Marine Research, Norway

## Abstract

Diving with sharks, often in combination with food baiting/provisioning, has become an important product of today’s recreational dive industry. Whereas the effects baiting/provisioning has on the behaviour and abundance of individual shark species are starting to become known, there is an almost complete lack of equivalent data from multi-species shark diving sites. In this study, changes in species composition and relative abundances were determined at the Shark Reef Marine Reserve, a multi-species shark feeding site in Fiji. Using direct observation sampling methods, eight species of sharks (bull shark *Carcharhinus leucas*, grey reef shark *Carcharhinus amblyrhynchos*, whitetip reef shark *Triaenodon obesus*, blacktip reef shark *Carcharhinus melanopterus*, tawny nurse shark *Nebrius ferrugineus*, silvertip shark *Carcharhinus albimarginatus*, sicklefin lemon shark *Negaprion acutidens*, and tiger shark *Galeocerdo cuvier*) displayed inter-annual site fidelity between 2003 and 2012. Encounter rates and/or relative abundances of some species changed over time, overall resulting in more individuals (mostly *C. leucas*) of fewer species being encountered on average on shark feeding dives at the end of the study period. Differences in shark community composition between the years 2004–2006 and 2007–2012 were evident, mostly because *N. ferrugineus*, *C. albimarginatus* and *N. acutidens* were much more abundant in 2004–2006 and very rare in the period of 2007–2012. Two explanations are offered for the observed changes in relative abundances over time, namely inter-specific interactions and operator-specific feeding protocols. Both, possibly in combination, are suggested to be important determinants of species composition and encounter rates, and relative abundances at this shark provisioning site in Fiji. This study, which includes the most species from a spatially confined shark provisioning site to date, suggests that long-term provisioning may result in competitive exclusion among shark species.

## Introduction

Sharks are an important component of coral reefs, both in terms of the ecological role they play in reef ecosystems [1]–[3], and increasingly because of their value for shark-diving tourism [4], [5]. Watching sharks in their natural habitats, and hence diving with sharks, has become an important product of today’s recreational dive industry [6]. However, in order to guarantee shark sightings to paying customers, dive operators are often required to use food to reliably attract them to specific dive sites [7]. Whereas the effects of baiting (i.e. chumming) and supplemental food provisioning (i.e. actual feeding) on, for example, the behaviour and abundance of individual shark species are starting to become known [8]–[13], there is an almost complete lack of equivalent data from multi-species shark diving sites.

In the only two studies on multiple species available to date, Meyer et al. [14] suggest that increasing numbers of larger Galapagos sharks *Carcharhinus galapagensis* and tiger sharks *Galeocerdo cuvier* gradually excluded smaller sandbar sharks *Carcharhinus plumbeus* at a baited dive site in Hawaii. In a more recent study, Clarke et al. [15] found that, at a reef in the Red Sea where sharks have been baited for more than 12 years, initially grey reef sharks *Carcharhinus amblyrhynchos* outnumbered silky sharks *Carcharhinus falciformis*, but over a six-year period *C. falciformis* sightings increased almost 20-fold, while *C. amblyrhynchos* sightings decreased by more than 90%. Subsequently, the number of *C. falciformis* also declined considerably, leading the authors to suggest that declines were related to local fishing pressure rather than competitive exclusion [15].

**Figure 1:**
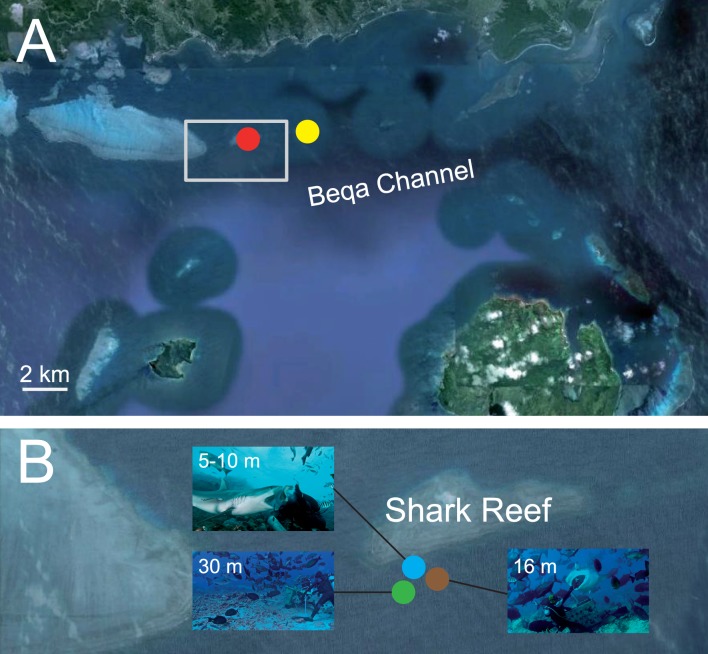
Study site on the southern coast of Viti Levu, Fiji. (A) Shark Reef (red dot) and Lake Reef (yellow dot); (B) feeding site at 30 m (green dot) where sharks and other fish are attracted at the beginning of the first dive of the day, a *C. amblyrhynchos* taking a fish fillet from the hand of the feeder at the shallow (5–10 m) feeding site (blue dot), and a *G. cuvier* taking a tuna fish head from the hand of the feeder at the 16 m feeding site (brown dot) on the second dive of the day.

In the present study, we evaluate data from the Shark Reef Marine Reserve, a multi-species shark feeding site in Fiji [16], [17]. Up to eight different species of sharks can be encountered at Shark Reef, namely bull sharks *Carcharhinus leucas*, whitetip reef sharks *Triaenodon obesus*, blacktip reef sharks *Carcharhinus melanopterus*, tawny nurse sharks *Nebrius ferrugineus*, silvertip sharks *Carcharhinus albimarginatus*, sicklefin lemon sharks *Negaprion acutidens*, *C. amblyrhynchos* and *G. cuvier*. Since 2003, parts of Shark Reef have been declared as a no-take zone that is visited 3 to 4 times per week by a single dive operator [17]. Previous research from this site has shown that the number of *C. leucas*, the numerically dominant species at the Shark Reef Marine Reserve, increased over the years, but decreased over the course of a calendar year [9], [16]. In 2006, a competitor dive operator started to conduct shark feeding dives on the neighbouring Lake Reef (Fig. 1A), mostly on the same days and times when dives on Shark Reef take place.

In order to determine changes in species composition and relative abundances at the Shark Reef Marine Reserve, we asked the following questions: 1) Did species composition and/or encounter rates change over time at the Shark Reef Marine Reserve? 2) Are there seasonal and/or long-term changes in relative abundance of the different shark species at the feeding site? 3) Does the presence of a competitor operator conducting shark feeds at a nearby reef have an effect on shark abundance at the Shark Reef feeding site? In answering these questions, our results provide baseline data on the long-term trends in relative abundance and seasonal cycles, and help elucidate whether the numbers of the eight species of sharks visiting the Shark Reef Marine Reserve changed over the years. Additionally, our data provide important fisheries-independent information on shark populations that can supplement long-term monitoring and serve for conservation purposes.

## Materials and Methods

### Ethics Statement

Field work was carried out in the Shark Reef Marine Reserve. No animals were caught or handled. All research methods were approved and conducted under a permit provided by the Fijian Ministry of Fisheries and with the knowledge and permission of the traditional owners of Shark Reef.

### Study Site and Data Collection Protocol

A single dive operator has exclusive access to the Shark Reef Marine Reserve, located on the southern coast of Viti Levu [17], where it hand-feeds sharks at three feeding sites about 10–30 m from one another at different depths (Fig. 1). The main attraction of the shark dive is *C. leucas*, the most abundant species at this shark provisioning site in Fiji [9]. Since its establishment in 2003, up to eight species of sharks can be encountered at the marine reserve [16].

The dive procedure starts with a first dive to 30 m to attract sharks with fish scraps dispersed out of a bin and/or whole fish heads (Fig. 1B). Here, only *C. leucas* and *N. ferrugineus* turn up regularly. Whereas the former species will only approach if whole fish heads are offered, *N. ferrugineus*, if present, persistently approach the person feeding sharks and/or the bin to feed on fish scraps and/or whole fish heads. A notable observation is that *C. leucas* will not approach the feeder if *N. ferrugineus* beleaguer the person and/or the bin. After 17 min, the divers ascend up the reef slope to the shallow water feeding site where the feeder hand-feeds *T. obesus*, *C. melanopterus* and *C. amblyrhynchos* with fish scraps for the remainder of the dive (∼20 min). After a one hour surface interval, a second dive is conducted at 16 m where the feeder hand-feeds the larger shark species that include *C. leucas*, *C. albimarginatus*, *N. acutidens* and *G. cuvier* with whole fish heads (mainly tuna) for ∼35 min (Fig. 1B).

Food amounts introduced daily were measured for the years 2009 and 2010. In 2009 (n = 169 sampling days) and 2010 (n = 164 sampling days), between 100 and 250 kg and 100 and 300 kg of fish, respectively, were each introduced on the first (mean_2009_±SD = 147.3±19.2 kg; mean_2010_±SD = 132.1±42.8 kg) and second dive of the day (mean_2009_±SD = 170.2±29.5 kg; mean_2010_±SD = 140.5±49.9 kg). Whole fish heads fed to sharks present at the 30 m and 16 m feeding sites were mainly *Thunnus* spp. (73 heads weighted; mean±SD = 2.42±0.85 kg; range = 1.5–6 kg). It is important to note that not all food introduced was consumed by sharks, but also other predatory fish such as giant trevally *Caranx ignobilis* and twinspot snapper *Lutjanus bohar* took bait, especially at the 30 m feeding site.

Data on species composition and relative abundances, measured as the number of individuals sighted per sampling day, were collected between January 2003 and June 2012 using direct observation sampling methods [9], [18]. A few trained observers including one of us (JMB) accompanied the commercial shark watching dives to collect the following data: 1) presence of each shark species (1373 sampling days; mean±SD = 137.3±34.2 days per year; Table S1), and 2) number of *C. leucas, C. amblyrhynchos*, *N. ferrugineus*, *T. obesus*, *C. melanopterus*, *C. albimarginatus*, *N. acutidens* and *G. cuvier* between January 2004 and June 2012. The number of male and female sharks was determined based on the presence or absence of claspers.

The only species that can be regularly encountered and fed on both dives of the day is *C. leucas*. They gradually show up on the first dive of the day at 30 m, and in lower numbers compared to the second dive of the day at 16 m [13]. Since we were interested in the maximum number of sharks present each day, and in order to avoid pseudoreplication and autocorrelation issues, we included the dive with the higher number of *C. leucas* in the analyses which was typically the second dive of the day [13].

### Data Analyses

The impact of food provisioning on species composition and shark relative abundance was estimated by conducting the dive protocol described above on five additional days in March 2008 during which no food was in the water. At all three feeding sites, the species present were recorded, the individuals counted and data were compared to data collected on feeding dives conducted in the same month (n = 8) with *t*-tests.

Encounter rates of each species were calculated for each year by dividing the number of days on which at least one individual of a given species was encountered by the total number of sampling days. A value of 1 indicates that the species was encountered on every sampling day. Sex-ratios were calculated by dividing the number of females encountered over the course of the study by the number of males; hence a value >1 indicates a sex-ratio skewed towards females, and a value <1 a sex-ratio skewed towards males.

Differences in shark community composition between years were tested using a two-way nested analysis of similarity (ANOSIM) based on relative abundance data, with months nested within years. Prior to the analysis, data were fourth-root transformed to reduce the dominance of dominant species, and hence make the highly abundant species less influential for the final ANOSIM result. Indeed, given the large differences in abundance of the different species, if no transformation was used the highly abundant species would most likely appear as the ones responsible for the differences. By transforming the data and reducing their importance, we make sure that the less abundant species also contribute to differences in community composition. The fourth-root transformed data was used to construct a Bray-Curtis similarity matrix, on which the ANOSIM was based. The year 2012 was not included in the analysis as data for this year was only available until June.

A multivariate classification and regression tree analysis (CART) [19] was also used to further explore differences and similarities of community composition between years and months, and to identify which species contribute the most to these differences. CART analysis is a robust non-parametric test that accommodates unbalanced designs, reduced sample sizes, missing values and outliers [19]. CARTs can also capture relationships such as non-linear effects that are difficult to resolve with conventional statistics [20]. CARTS successively partition the dataset into two relatively homogeneous and mutually exclusive groups, based on a single explanatory variable. Since in the present study the dependent variable (number of sharks sighted) is continuous, splitting is based on minimising the within-group sum of square residual deviation of the resultant groups. Trees are represented in a graphical way, with the root node on top, representing the initial assemblage of data, from which the branches and leaves emerge. Splits close to the root node are more important than those closer to the bottom of the tree, providing greater improvement to the fit of the model. The proportion of the total sum of squares explained by each split is indicated by the relative lengths of the vertical lines associated with each split. The size of the tree is selected by 10-fold cross validation, and the 1-SE tree, i.e. the smallest tree within 1 standard deviation of the three with the lowest CV-error, was considered the final tree model. This analysis provides statistically robust and ecologically meaningful interpretations of data [19], [20].

Because most species often occurred in low numbers, including zeros, and to reduce variability, input data consisted of the number of sharks detected per 3-day blocks, after the days within each month were placed in a random order to avoid potential autocorrelation of data. Data was fourth-root transformed prior to this analysis. CARTS were also used to identify the effect of year and month on the relative abundance of each shark species separately so that any trend or seasonality in abundance could be identified. For *C. leucas*, input data was the number of sharks sighted per day, as this species was highly abundant and present on almost every dive. For the remaining species, however, input data consisted of the number of sharks detected per 3-day blocks, after the days within each month were placed in a random order, as explained above. Analyses were done using the TREES package on S-PLUS 2000® (MathSoft, Cambridge, MA, USA).

### Competitor Operator

From 2006 onwards, a competitor dive operator conducted shark feeding dives on the neighbouring Lake Reef (Fig. 1A; distance between Shark Reef and Lake Reef is ∼3 km) mostly on the same days and times when dives on Shark Reef took place. The competitor operator at Lake Reef, to the best of our knowledge, does not collect similar data that could form the basis for a comparison with data collected at Shark Reef. Nevertheless, in order to assess the potential influence shark feeding on Lake Reef has on species composition and shark numbers at the Shark Reef Marine Reserve, we examined our shark counts on days with and without competitor feeds. To determine if the competitor’s operation had an effect on the abundance of sharks at the Shark Reef feeding site, ANOVAs were used to test for differences in number of sharks sighted at Shark Reef between January 2009 and June 2012 (n = 524 sampling days) between the days that the competitor conducted shark feeds (n = 383 days) and the days it did not (n = 141 days). This was only done for the four most abundant species: *C. leucas*, *C. amblyrhynchos*, *T. obesus* and *C. melanopterus*. For *C. leucas* and *C. amblyrhynchos*, the two most abundant species at the Shark Reef Marine Reserve, only data for the months of greatest abundance, as identified by the CARTS, were considered: January to August for *C. leucas*, and July to December for *C. amblyrhynchos*. For the remaining, least abundant species, the generally smaller number of sightings (including many zeroes) did not allow for robust ANOVAs, and therefore only anecdotal evidence is presented.

## Results

### Species Composition and Encounter Rates

**Figure 2:**
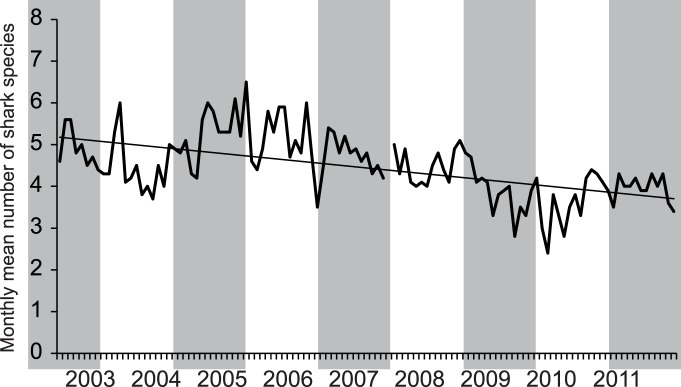
Monthly mean number of species encountered at the Shark Reef Marine Reserve per sampling day between January 2003 and June 2012. No data are available for January 2008. The mean number of species was calculated by dividing the number of species counted per day by the number of sampling days in the respective month.

Eight species of sharks were recorded, with overall encounter rates ranging from 0.09 for *G. cuvier* to 0.99 for *C. leucas* (Table 1). The number of species encountered on a single sampling day in the Shark Reef Marine Reserve decreased over the course of the study (y = −0.131x+5.223, *R* ^2^ = 0.303, *F*_(1,109)_ = 47.284, *p*<0.0001) (Fig. 2). With the exception of *C. albimarginatus* and *G. cuvier* in 2012, all species were encountered in all years (Table 1). There was an effect of year (ANOSIM, *R = *0.353, *p = *0.001) and month (*R = *0.206, *p = *0.001) on shark species composition. Significant differences in species composition were found between 22 out of the 28 possible pairs of years (Table 2). The only pair of consecutive years between which there was no significant change in community composition was 2008–2009. See CART results below for the drivers of these differences.

**Table 1:** Encounter rates of each shark species at the Shark Reef Marine Reserve.

Species	Encounter rate										
	2003	2004	2005	2006	2007	2008	2009	2010	2011	2012	Mean (±SD)
*Carcharhinus leucas*	0.99	0.99	0.99	0.96	1.00	1.00	1.00	1.00	1.00	1.00	0.99 (0.01)
*Carcharhinus amblyrhynchos*	0.95	0.88	0.94	0.97	0.96	0.98	0.96	0.88	0.91	0.84	0.93 (0.05)
*Triaenodon obesus*	0.42	0.25	0.70	0.92	0.98	0.97	0.93	0.69	0.96	0.94	0.77 (0.26)
*Carcharhinus melanopterus*	0.57	0.47	0.71	0.76	0.90	0.79	0.77	0.62	0.94	0.91	0.74 (0.15)
*Nebrius ferrugineus*	0.90	0.77	0.90	0.78	0.56	0.37	0.34	0.10	0.25	0.24	0.52 (0.30)
*Carcharhinus albimarginatus*	0.62	0.62	0.47	0.32	0.06	0.06	0.05	0.01	0.03	0.00	0.22 (0.26)
*Negaprion acutidens*	0.31	0.35	0.44	0.29	0.18	0.06	0.05	0.03	0.03	0.01	0.18 (0.16)
*Galeocerdo cuvier*	0.12	0.09	0.16	0.15	0.10	0.14	0.05	0.05	0.03	0.00	0.09 (0.05)

**Table 2:** ANOSIM results, including *R*- (top of matrix) and *p*-values (bottom of matrix) related to the comparisons of community composition between pairs of years.

	2004	2005	2006	2007	2008	2009	2010	2011
2004		0.254	0.534	0.74	0.757	0.777	0.884	0.852
2005	**0.011**		0.069	0.346	0.474	0.462	0.765	0.632
2006	**0.001**	**0.045**		0.126	0.252	0.244	0.639	0.507
2007	**0.001**	**0.001**	**0.070**		0.106	0.094	0.501	0.403
2008	**0.001**	**0.001**	**0.001**	**0.035**		0.000	0.330	0.340
2009	**0.001**	**0.001**	**0.001**	**0.033**	0.471		0.176	0.175
2010	**0.001**	**0.001**	**0.001**	**0.001**	**0.001**	**0.001**		0.151
2011	**0.001**	**0.001**	**0.001**	**0.001**	**0.001**	**0.002**	**0.002**	

Significant results are in bold.

**Figure 3:**
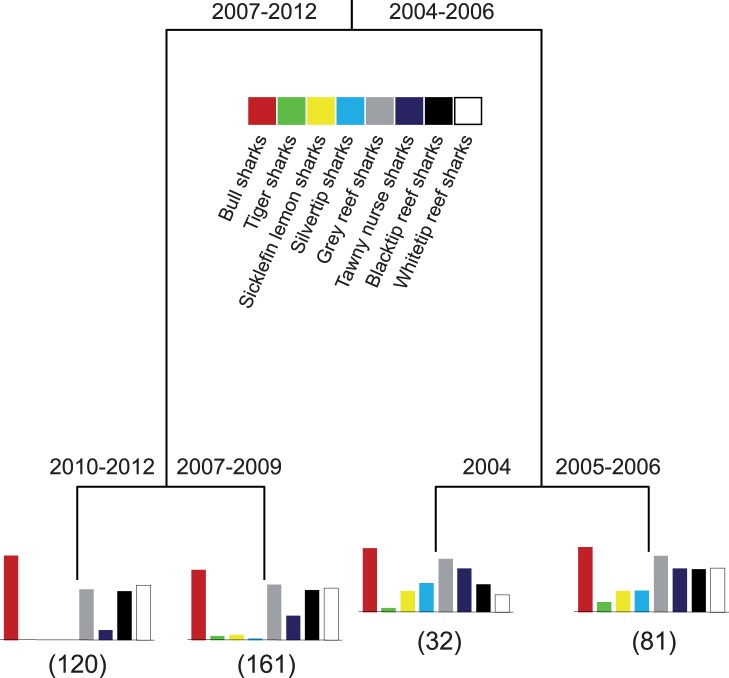
Four-leaf classification and regression tree explaining 32% of the total variability and showing differences in shark community composition between years and months based on relative abundance data from visual surveys. Bar graphs below terminal nodes indicate relative distribution of species abundance (based on fourth-root transformed data on number of sharks detected per 3-day blocks, after the days within each month were placed in a random order). Numbers in brackets are sample sizes. The colour code denotes the colour used in all figures for the respective species throughout this article.

**Figure 4:**
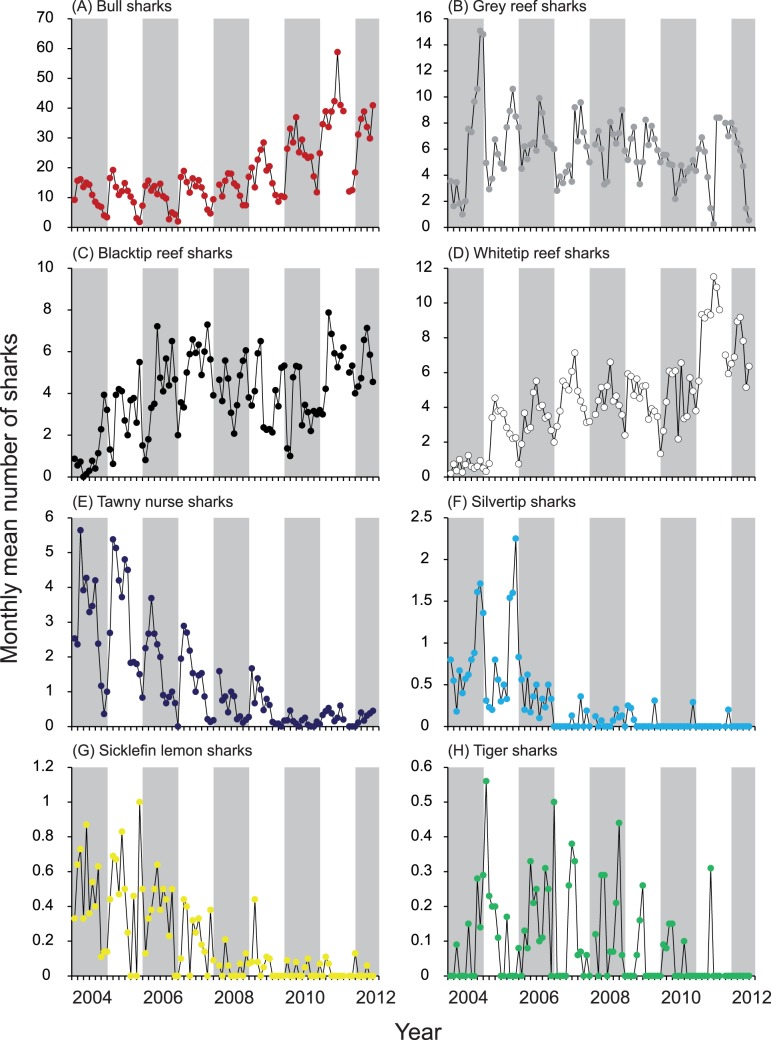
Monthly mean number of sharks sighted per sampling day between January 2004 and June 2012. The mean number of sharks was calculated by dividing the number of individuals counted per day by the number of sampling days in the respective month.

The CART analysis also detected significant differences in shark community composition between years (Fig. 3). Although a 12-leaf tree, explaining 42% of the variability was most often selected as the final model according to the 1-SE rule, this leads to a very complex and hard to interpret tree, with the less important branches contributing little improvement to the fit of the model (<3% each). So, we present a simpler 4-leaf tree, which contains the most important splits and explains 32% of the model (Fig. 3). This tree indicates that shark community composition differed the most between the block of years of 2004–2006 and 2007–2012 (Fig. 3). This split explained 24% of the total variability; *N. ferrugineus* (7.1%) and *C. albimarginatus* (6.2%) contributed the most to this difference, followed by *N. acutidens* (3.8%) and *T. obesus* (3.3%). The reasons for this importance can be seen in Fig. 4, where it is clear that *N. ferrugineus*, *C. albimarginatus* and *N. acutidens* were more abundant in 2004–2006 and rare in the period of 2007–2012, whereas for *T. obesus* the pattern was the opposite: more individuals were sighted between 2007 and 2012 than between 2004 and 2006. Secondary splits on the CART indicate further differences between 2004 and 2005–2006, and between 2007–2009 and 2010–2012 (Fig. 3). Overall, these differences were mostly driven by *N. ferrugineus* (9.2%), followed by *C. albimarginatus* (6.5%), *T. obesus* (5.8%), *N. acutidens* (4.1%) and *C. leucas* (3.6%). *Carcharhinus amblyrhynchos* (0.3%) and *G. cuvier* (0.7%) had almost no influence in the separations, as their numbers were similar throughout the study period (Fig. 4B and H).

### Relative Abundances and Sex Ratios

The most abundant species throughout the study was *C. leucas*, whereas *G. cuvier* was the least abundant, with a mean number of sharks recorded per day for a given month often <0.1 (Fig. 4). *Galeocerdo cuvier*, *C. albimarginatus* and *N. acutidens* were encountered in low numbers, with usually only one individual present (maximum numbers present: *G. cuvier* = 2, *C. albimarginatus* = 3, *N. acutidens* = 3). All *G. cuvier* and *C. albimarginatus* encountered at the Shark Reef feeding site were females. Also, all *N. acutidens* were females, with the exception of three days each with one male individual present. Similar to *C. leucas* (3.28), *T. obesus* showed a sex ratio skewed towards females (2.42), whereas *C. melanopterus* (0.91), *C. amblyrhynchos* (0.78) and *N. ferrugineus* (0.04) had sex-ratios skewed towards males.

**Figure 5:**
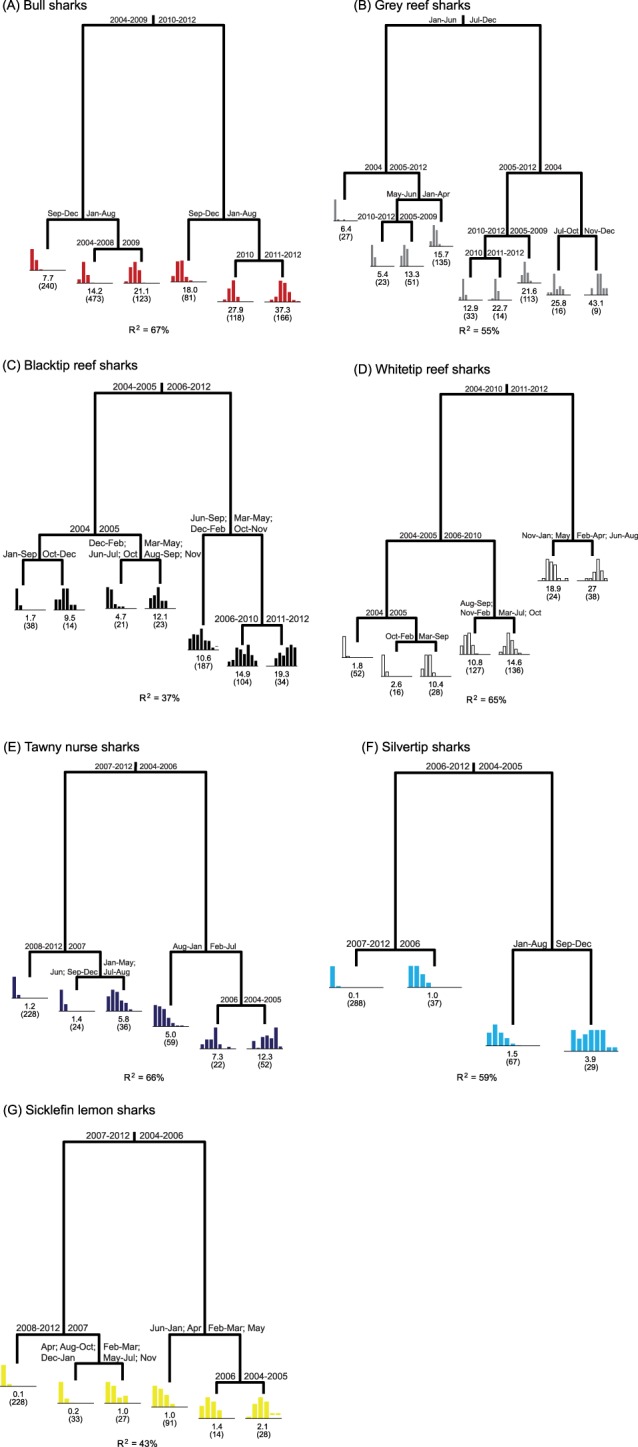
Classification and regression trees explaining shark relative abundance based on year and month. For *C. leucas*, trees were constructed using the number of individuals sighted per day. For the remaining species, due to the often low numbers, analyses were done based on the number of sharks detected per 3-day blocks, after the days within each month were placed in a random order. Histograms of distribution of relative abundance are presented below the terminal nodes, and mean number and sample size (in brackets) are also indicated.

There were differences in trend and seasonality in shark sightings between species (Figs. 3 and 4). For *C. leucas*, a six-leaf tree, explaining 67% of the variability, indicates that the number of sharks sighted per day increased through time, as significantly fewer sharks were sighted in the years of 2004–2009 than between 2010 and 2012 (Figs. 3A and 4A). For both branches of the tree, secondary splits indicate seasonality in shark abundance, with more sharks present between January and August, and fewer sharks sighted between September and December (Fig. 5A). For the months of highest abundance, further splits again indicate an increasing trend in *C. leucas* abundance between 2004 and 2012.

For *C. amblyrhynchos*, there was no trend in shark numbers through time (Figs. 3B and 4B), and month was the most important variable explaining shark abundance (Fig. 5B), indicating strong seasonality. Overall, more sharks were sighted per day between July and December than between January and June (Fig. 5B). For the months of higher abundance, more sharks were present between 2005 and 2012 than in 2004, suggesting an initial increase in shark abundance. This can also be seen in Fig. 4B. Further splits by both months and years were present, but these explain very little additional variability.

For *C. melanopterus*, as with *C. leucas*, abundance increased through time, as significantly more sharks were sighted between 2006 and 2012 than between 2004 and 2005 (Figs. 3C and 4C). On average, 2004 had the lowest number of sharks sighted, and 2011–2012 the highest. For the 2006–2012 period, the number of sharks sighted was higher in the months of March–May and October–November than in June–September and December–February. Overall, the effects of year and month explained 37% of the variability in number of *C. melanopterus* sighted (Fig. 5C).

For *T. obesus*, there was also an increase in shark relative abundance through time (Figs. 3D and 4D). More sharks were sighted between 2011 and 2012, followed by 2006–2010, 2005, and 2004 was the year when the smallest number of *T. obesus* was sighted (Fig. 5D). For the years of 2011 and 2012, there was a small seasonal effect, as more sharks were sighted between February and August, and fewer sharks between November and January (Fig. 5D). Note that May was classified together with the November–January group, likely due to small sample size and high variability in the sightings.

For *N. ferrugineus*, there was a decrease in number of sightings from 2004 to 2012 (Figs. 3E and 4E). Significantly more sharks were sighted between 2004 and 2006 than between 2007 and 2012, with lowest numbers recorded between 2008 and 2012, and highest between 2004 and 2005 (Fig. 5E). For data between 2004 and 2006, there was also some seasonality in shark abundance, as more sharks were generally sighted between February and July than between August and January (Fig. 5E). Similarly, for 2007, there was some seasonality in shark numbers, but this explained little of the variability.

Similar to *N. ferrugineus*, there was a decrease in abundance of *C. albimarginatus* through time (Figs. 3F and 4F). Significantly more *C. albimarginatus* were sighted between 2004 and 2005 than between 2006 and 2012, with the lowest numbers recorded between 2007 and 2012 (Fig. 5F). For the years of higher abundance (2004 and 2005), there was a seasonality in *C. albimarginatus* abundance, as more sharks were sighted per day between September and December than between January and August (Fig. 5F). Overall, the effect of year and month explained 59% of the variability in the number of *C. albimarginatus* sighted.

For *N. acutidens*, as with *C. albimarginatus* and *N. ferrugineus*, the number of individuals sighted decreased from 2004 to 2012 (Figs. 3G and 4G). Significantly more sharks were present between 2004 and 2006 than between 2007 and 2012, and this year separation explained most of the variability (Fig. 5G). For the years 2004 to 2006, a secondary split suggests a seasonal effect, as *N. acutidens* were more abundant between February and May than between June and January. Although April was grouped with the June–January group, that is most likely a misclassification due to small number of samples or high variability between groups. For the months with higher abundances, more sharks were sighted in 2004 and 2005 than in 2006, and for the 2007–2012 branch, a secondary split indicates that fewer sharks were sighted in 2007–2012 than in 2007 (Fig. 5G), again indicating a decrease in numbers through time.

For *G. cuvier*, CART analysis did not reveal any effect of year or month in shark abundance, as neither of these variables was responsible for a branch separation in the tree. Thus, no trend or seasonality in *G. cuvier* numbers was present (Fig. 4H).

### Comparison of Species Composition and Shark Relative Abundance between Feeding and Non-feeding Days (March 2008)

**Figure 6:**
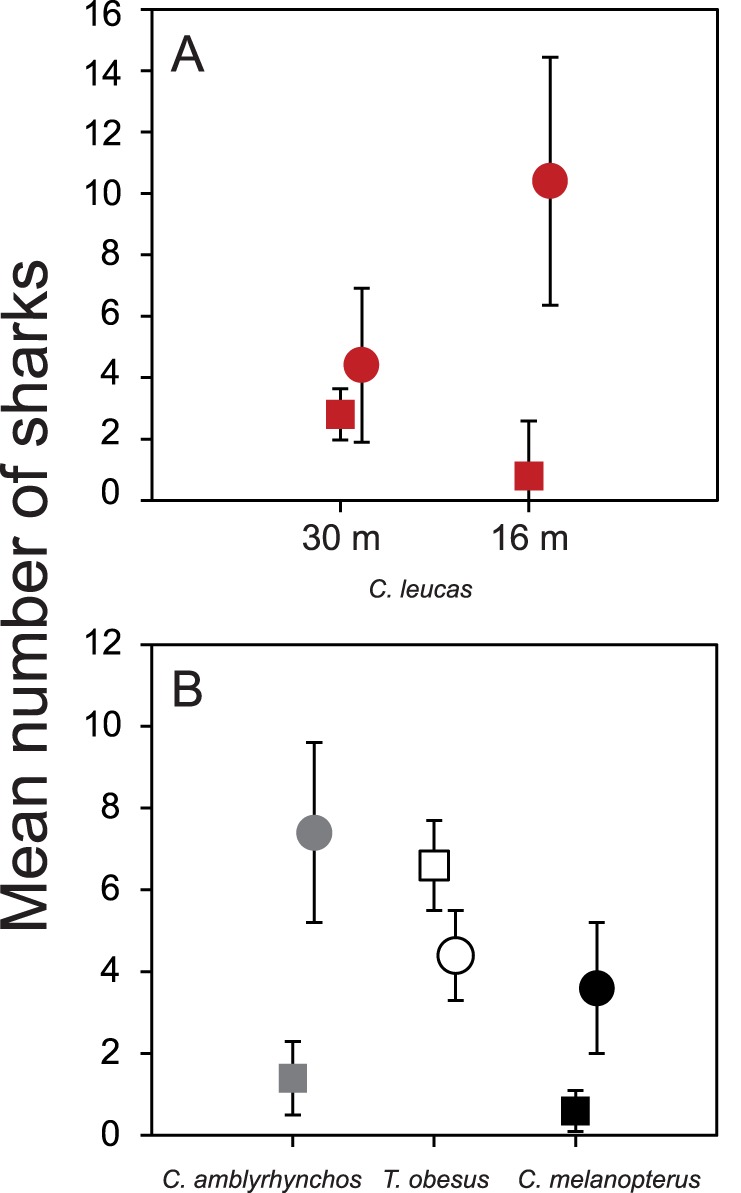
Mean number of sharks counted on feeding and non-feeding dives in March 2008. (A) Mean (±SD) number of *C. leucas* counted at the 30 m feeding site and at the 16 m feeding site, and (B) mean (±SD) numbers of *C. amblyrhynchos*, *T. obesus* and *C. melanopterus* counted at the shallow (5–10 m) feeding site. Circles denote sampling days on which sharks were offered food and rectangles denote non-feeding days.

Overall, five shark species (*C. leucas*, *C. amblyrhynchos*, *N. ferrugineus*, *C. melanopterus* and *T. obesus*) were recorded at the Shark Reef Marine Reserve in March 2008, both on days when food was in the water and when not. On days when no food was in the water, all sharks were recorded when descending and disappeared out of sight after a few minutes, without approaching the divers. The numbers of *C. leucas* sighted at the 30 m feeding site did not differ between feeding and non-feeding days (t_10_ = −1.382, p>0.05; Fig. 6A), but significantly fewer *C. leucas* were encountered at the 16 m feeding site when no food was in the water (t_11_ = −4.949, p<0.001; Fig. 6A). At the shallow (5–10 m) feeding site, significantly more *T. obesus* (t_11_ = 3.580, p<0.05) and fewer *C. amblyrhynchos* (t_11_ = −5.709, p<0.001) and *C. melanopterus* (t_11_ = −2.512, p<0.05) were encountered when no food was in the water (Fig. 6B).

### Competing Operator Presence

There were no significant differences in relative abundance estimates of *C. leucas*, *C. amblyrhynchos*, *T. obesus* or *C. melanopterus* at the Shark Reef Marine Reserve between days when the competitor operator conducted feeding dives at Lake Reef and days it did not (*C. leucas* (peak abundance periods only): ANOVA, *F* = 2.936, *p* = 0.087; *C. amblyrhynchos* (peak abundance periods only): *F* = 0.0286, *p* = 0.865; *T. obesus*: *F* = 0.515, *p* = 0.473; *C. melanopterus*: *F* = 0.213, *p* = 0.473).

## Discussion

Our findings show a dynamic and variable picture clearly indicating that not all shark species that visit Shark Reef Marine Reserve show similar responses to provisioning, and that the responses can also change over time. All eight species in this study displayed inter-annual site fidelity, but encounter rates and/or relative abundances of some species changed over time, overall resulting in more individuals of fewer species having been encountered on average on shark feeding dives at the Shark Reef Marine Reserve at the end of the study period. We offer two explanations for the observed changes in relative abundances over time, namely inter-specific interactions and operator-specific feeding protocols. Both, possibly in combination, are suggested to be important determinants of species composition and encounter rates, and relative abundances at this shark provisioning site in Fiji.

Relative abundances of *T. obesus* and *C. melanopterus* increased whereas *C. amblyrhynchos*, the second most abundant species at Shark Reef [16], showed no trend in numbers through time (Fig. 4B–D). Although it is unknown to what extent and how these three smaller reef shark species used Shark Reef before the feeding operation started, our findings can best be explained with species-specific degrees of site fidelity and size of home ranges. Although capable of longer range movements [21]–[23], large-scale movements are thought to be comparatively limited in the three species of reef sharks, with pronounced long-term site fidelity to specific small-scale coastal or reef habitats [22]–[27]. It is therefore possible that at least some individuals from the three smaller reef shark species were already using the reef before the feeding operation started and additional individuals were attracted gradually from other reefs in the area.

Despite year-round availability of food, individual sharks [13] and the different species were not permanently entrained to the Shark Reef Marine Reserve. Of the four most abundant species encountered, *C. leucas*, *C. amblyrhynchos* and *C. melanopterus* showed seasonal trends throughout the study, whereas for *T. obesus* a small seasonal effect was only found for the years of 2011 and 2012. Seasonal trends in relative abundance are known both from sites where elasmobranchs naturally aggregate [22], [26], [28]–[30], and sites to which sharks are attracted for tourism [8], [12], [14]. Such trends in abundance have been suggested to relate to reproductive activity [9], [12], [14], [22], [29].

It is plausible that competition, both among and within species, which is known to influence the distribution of sharks over relatively small spatial scales [31]–[33], determines species composition and relative abundances at multi-species provisioning sites. In this sense, the larger species, namely *C. leucas* in this case, competitively exclude the smaller reef shark species from certain feeding sites at the Shark Reef Marine Reserve [34]. This hypothesis may explain the finding that the smaller reef sharks species were never observed approaching the feeder at the 30 m and 16 m feeding sites where the larger species are fed.

The possible existence of dominance hierarchies [35] among shark species provisioned at the Shark Reef Marine Reserve may also explain the decreasing relative abundances of *C. albimarginatus* and *N. acutidens* at the 16 m feeding site. Analysing data from 2003 to 2009, Brunnschweiler & Baensch [9] showed that *C. leucas* relative abundance at Shark Reef increased since regular feeding began in 2003, and speculated that this increase might have affected abundance, encounter rates and/or the behaviour of other shark species at the site. Including data from January 2010 to June 2012 in the current study, we found the increasing trend in *C. leucas* relative abundance to continue (Fig. 4A). Hence, the observed trends and patterns may be a localised, behaviourally-mediated phenomenon, with *C. leucas* increasing in numbers ([9]; this study), gradually excluding other species from the feeding site [14].

Anecdotal observations from the Shark Reef Marine Reserve indicate that, as numbers of *C. leucas* increased over the years ([9]; this study), *C. albimarginatus* and *N. acutidens* were less able to approach the feeder. It is reasonable to assume that sharks regularly return to provisioning sites only if they receive a reward upon their visit, which got increasingly difficult over the years due to the increasingly larger number of *C. leucas* at the site. The same mechanism may explain the decrease in *N. ferrugineus* numbers after 2006 (Fig. 4E), as these were literally chased away from the feeding site by the dive operator. But *C. leucas* preventing other shark species from obtaining sufficient reward to make their return worthwhile is only one potential mechanism of exclusion. Another mechanism could be inter-specific aggression. However, this was never observed at the Shark Reef Marine Reserve.

Virtually no empirical information is available on the effects of several shark feeding companies operating simultaneously at the same site or close to each other on species composition and relative abundances [14]. Our results, in combination with anecdotal information, allow us to speculate that the different feeding protocol used by the competitor operator at Lake Reef had an effect on relative abundances of at least some of the least abundant species that can be encountered at the Shark Reef Marine Reserve. Whereas the ANOVAs did not find significant differences in relative abundance for the four most prevalent species (*C. leucas*, *C. amblyrhynchos*, *T. obesus* or *C. melanopterus*) at the Shark Reef Marine Reserve between days when the competitor operator conducted feeding dives at Lake Reef and days it did not, the less prevalent species *N. ferrugineus*, *C. albimarginatus* and *N. acutidens* became very rare in the period 2007–2012 (Fig. 4E–G), the time period during which the competitor operator offered food on Lake Reef. It is therefore possible that, in combination with the possibly existing competitive exclusion at the Shark Reef feeding sites (see above), individuals of the latter three species gradually moved from the Shark Reef Marine Reserve to Lake Reef.

This interpretation is supported by anecdotal information provided by several SCUBA divers who dived both Shark Reef and Lake Reef, particularly one individual who attended 368 shark feeding dives at both reefs (75% at Lake Reef) on a continuous basis between 2008 and 2012, and their description of the diving and feeding protocols conducted by the competitor operator. Here, the relative number of *C. albimarginatus* was reported to have increased since 2008, when it was usual to see one or two large *C. albimarginatus* compared to often >10 in 2012. On the other hand, the number of *C. leucas* encountered at Lake Reef was reported to have dropped over time, while *G. cuvier* were reported to turn up more often and in higher numbers at Lake Reef compared to the Shark Reef feeding site. On several dives between 2010 and 2012 there were multiple *G. cuvier* (maximum = 3) at the Lake Reef feeding site at the same time.

The feeding protocols differed between the two dive sites, as the operator at Lake Reef dumps bins of “fish loins” consisting of fish offcuts and skin sporadically during the dive, and also hand-feeds tuna heads to sharks. When fish loin dumping starts, large numbers of *N. ferrugineus* (>20) and several *N. acutidens* and *C. leucas* show up at the feeding site. *Carcharhinus albimarginatus* turn up generally within 10 min after the fish loins are released.

With the increasing number of shark diving sites around the globe [6], opportunities for investigating the effects food provisioning has on the behaviour and ecology of the target animals have increased. Unfortunately, there is still an almost complete lack of information from multi-species shark diving sites [14], [15]. Our study, which includes the most species from a spatially confined shark provisioning site to date, contributes by starting to fill this critical knowledge gap. However, despite the increasing amount of information available about relative abundances, movement patterns and behaviour of sharks at the Shark Reef Marine Reserve ([9], [13], [17]; this study), many open questions remain. For example, it is not known how shark provisioning influences the community ecology and ecosystem dynamics in the area around Shark Reef. Effects such as changing natural community composition, species richness and/or increased predation pressure are known, at least to some extent, from teleost provisioning sites [36]–[39].

We strongly encourage shark diving operators and academics to team up and implement long-term research programmes at provisioning sites to monitor sharks’ responses to food provisioning and its effects on adjacent communities and ecosystems. Additionally, long-term monitoring of sharks and other marine wildlife at such sites that are often located in protected areas will provide much needed temporal data on population sizes that will benefit conservation efforts and protected area management [40]–[42]. We hope that our study will serve as a template for such collaborations.

## Supporting Information

Table S1 Number of sampling days per month per year.
(PDF)
