# Correction: Long-Term Changes in Species Composition and Relative Abundances of Sharks at a Provisioning Site

**DOI:** 10.1371/journal.pone.0094148

**Published:** 2014-04-04

**Authors:** The PLOS ONE Staff

There are formatting errors in the text of the PDF and in Figure 2 that were introduced while the article was being prepared by production for publication. The journal apologizes for these errors and is including the correct text and figure below.

In the second sentence of the fourth paragraph of the Materials and Methods section, there are incorrect line breaks. The correct sentence should read:

In 2009 (n  =  169 sampling days) and 2010 (n  =  164 sampling days), between 100 and 250 kg and 100 and 300 kg of fish, respectively, were each introduced on the first (mean2009 ± SD  =  147.3 ± 19.2 kg; mean2010 ± SD  =  132.1 ± 42.8 kg) and second dive of the day (mean2009 ± SD  =  170.2 ± 29.5 kg; mean2010 ± SD  =  140.5 ± 49.9 kg).

The grey shaded areas in Figure 2 are too large. Please see the correct Figure 2 below.

**Figure 2:**
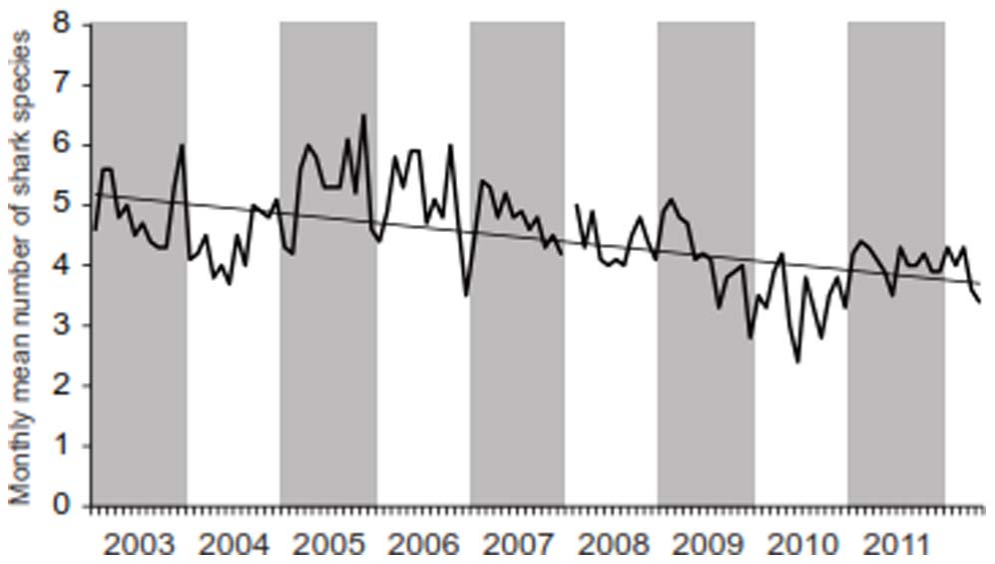
Monthly mean number of species encountered at the Shark Reef Marine Reserve per sampling day between January 2003 and June 2012. No data are available for January 2008. The mean number of species was calculated by dividing the number of species counted per day by the number of sampling days in the respective month.
