# Supplementary material for: Long-Term Changes in Species Composition and Relative Abundances of Sharks at a Provisioning Site
Source: PLoS One. 2014 Jan 23;9(1):e86682. doi: 10.1371/journal.pone.0086682 (PMC3900589; doi:10.1371/journal.pone.0086682)
Supplement: Table S1 — Number of sampling days per month per year. (PDF) [file pone.0086682.s001.pdf]

Table S1. Number of sampling days per month per year. NA = not available.

	January	February	March	April	May	June	July	August	September	October	November	December	Total
**2003**	5	8	9	5	7	10	9	10	9	12	10	7	101
**2004**	15	11	11	12	15	14	13	10	8	18	14	14	155
**2005**	16	13	15	15	18	10	8	6	13	5	4	12	135
**2006**	16	15	13	6	14	8	10	9	13	4	3	2	113
**2007**	21	9	10	17	19	16	15	17	14	14	16	11	179
**2008**	NA	17	8	14	17	14	15	14	14	9	16	15	153
**2009**	12	9	13	16	19	19	21	16	13	13	13	6	170
**2010**	11	13	13	13	11	17	20	20	15	13	14	15	175
**2011**	12	9	15	13	13	4	10	10	NA	1	15	8	110
**2012**	9	15	18	16	13	11							82
